# *Mycobacterium tuberculosis* Beijing Genotype, the Netherlands

**DOI:** 10.3201/eid0910.020743

**Published:** 2003-10

**Authors:** Martien W. Borgdorff, Petra de Haas, Kristin Kremer, Dick van Soolingen

**Affiliations:** * Royal Netherlands Tuberculosis Association, the Hague, the Netherlands; † University of Amsterdam, Amsterdam, the Netherlands; ‡ National Institute of Public Health and the Environment (RIVM), the Hague, the Netherlands

**Keywords:** *Mycobacterium tuberculosis*, epidemiology, multidrug resistance

## Abstract

To determine whether the Beijing genotype of *Mycobacterium tuberculosis* is emerging in the Netherlands, we collected data on 6,829 patients during 1993 to 2000. Six percent had the Beijing genotype. This genotype was associated with diagnosis in recent years, young age, nationality, and multidrug resistance.

The Beijing genotype is found frequently in Asia (*1*–*3*) but also in outbreaks of multidrug-resistant tuberculosis (MDRTB) in various parts of the world, including Cuba, Germany, Russia, and Estonia (*4*–*7*). The largest known epidemic of MDRTB in North America was caused by the W strain, a variant of the Beijing genotype (*8*,*9*). A recent study showed this strain’s emergence and association with drug resistance in Vietnam (*10*). The Beijing genotype was also responsible for a recent outbreak of tuberculosis (TB) on the Canary Islands (*11*).

The relatively high degree of genetic conservation of Beijing genotype strains found in a widespread area (*12*) suggests a recent dissemination and, hence, selective advantages associated with this genotype of *Mycobacterium tuberculosis*. Recently, Beijing genotype bacteria were reported to carry mutations in putative mutator genes, which may explain a higher adaptability of these bacteria under stress conditions such as exposure to the intracellular environment or anti-TB drugs (*13*). These mutations may also be the basis of differential interaction between Beijing genotype bacteria and the host immune defense system suggested in a recent study in Indonesia (*14*).

In the Netherlands, MDRTB is affecting <1% of new TB patients, in particular immigrants (*15*–*17*). The incidence of the Beijing genotype has not yet been described. The goal of our study was to determine whether the Beijing genotype is emerging in the Netherlands and whether this genotype is associated with multidrug resistance.

## The Study

Patient data were obtained from the Netherlands Tuberculosis Register, which is maintained by the Royal Netherlands Tuberculosis Association and has been in place since 1993. Municipal health services, which are responsible for the followup of all TB patients, send information using standardized, precoded forms on all reported TB cases to the register. The register includes information on demographic characteristics, case detection, risk groups, type of disease, treatment, and treatment outcome.

Isolates obtained from all 8,210 culture-positive patients underwent restriction fragment length polymorphism (RFLP)–typing with IS*6110* as a probe at the National Institute of Public Health and the Environment. The Beijing genotype was defined on the basis of spoligotype (no spacers 1–34; at least 4 of spacers 35–43) and a specific region A insertion (*18*). Certain IS*6110* RFLP-genotype families (clades 47 and 61) were consistently found to be Beijing genotype (*18*). Isolates were assigned to the Beijing genotype on the basis of the IS*6110* RFLP pattern (*18*). If any doubt about clade membership existed, we used spoligotyping for the final allocation.

The RFLP results for the period 1993–2000 were matched to patient data on the basis of date of birth, postal area code, and sex, resulting in a perfect match for 5,994 (73%) patients and a near-perfect match for 835 (10%) patients; overall, 83% of the samples matched (6,829 patients). During this period, the Beijing genotype was observed in 516 cultures, representing 6% of the 8,210 patients. In the matched dataset, 415 (6%) of 6,829 were of the Beijing genotype. The number of TB cases attributable to the Beijing genotype tended to increase over time (Figure; p>0.05). This tendency was observed both among Dutch patients (r=0.66, 95% confidence interval [CI] –0.08 to 0.93) and non-Dutch patients (r=0.59, 95% CI –0.20 to 0.91) (Figure). While the increase in the number of cases among immigrants may be associated with an overall increase of immigrants to the Netherlands during the study period, the increasing numbers among Dutch patients likely reflect an increasing incidence rate.

**Figure:**
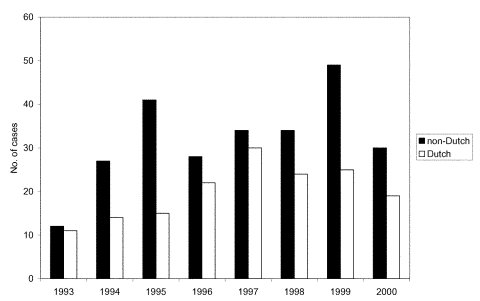
Number of tuberculosis cases with the Beijing genotype, Netherlands, 1993–2000.

The proportion of TB patients who had the Beijing genotype was significantly associated in univariate analysis with a later year of diagnosis, young age, nationality (increased among immigrants from Asia and decreased among those from Morocco, Turkey, Somalia, and other African countries, compared with Dutch citizens), and multidrug resistance (Table). These associations persisted in multivariate analysis (Table).

**Table:** Risk factors for the Beijing genotype of *Mycobacterium tuberculosis*, the Netherlands, 1993–2000

Risk factor	Beijing genotype	Total	(%)	Odds ratio	
				Crude	Adjusted^b^ (95% confidence intervals)
**Y of diagnosis**					
1993	23	669	3	1.09	1.08 (1.03 to 1.13)
1994	41	941	4	(per year)	
1995	56	863	6		
1996	50	946	5		
1997	64	872	7		
1998	58	804	7		
1999	74	879	8		
2000	49	855	6		
**Sex**					
Male	254	4,135	6	1	
Female	161	2,694	6	0.97	
**Age group**					
<25	120	1,522	8	1	1
25–34	123	1,925	6	0.80	0.69 (0.53 to 0.91)
35–44	64	1,080	6	0.74	0.60 (0.43 to 0.83)
45–54	23	614	4	0.45	0.36 (0.23 to 0.57)
55–64	24	493	5	0.60	0.55 (0.35 to 0.88)
65–74	40	517	8	0.98	0.78 (0.52 to 1.15)
75+	21	678	3	0.37	0.30 (0.18 to 0.50)
**Nationality**					
Netherlands	160	2,825	6	1	1
Europe (central and eastern)	20	231	9	1.58	1.27 (0.77 to 2.09)
Turkey	3	339	1	0.15	0.12 (0.04 to 0.38)
Morocco	19	650	3	0.50	0.41 (0.25 to 0.68)
Somalia	21	957	2	0.37	0.27 (0.17 to 0.44)
Africa (other)	28	594	5	0.82	0.63 (0.41 to 0.96)
Asia	140	786	18	3.61	3.01 (2.32 to 3.91)
Other	19	345	6	0.97	0.86 (0.53 to 1.42)
Unknown	5	102	5	0.86	0.73 (0.29 to 1.84)
**RFLP clustering^a^**					
No	212	3,227	7	1	
Yes, first case	57	1,052	5	0.81	
Yes, later case	146	2,550	6	0.86	
**Localization**					
Pulmonary	268	4,064	7	1	
Extrapulmonary	113	2,122	5	0.80	
Pulmonary and extrapulmonary	34	643	5	0.79	
**Residing in Netherlands**					
<6 months	46	641	7	1	
6–11 mo	12	301	4	0.54	
12–23 mo	28	358	8	1.10	
2–4 y	53	767	7	0.96	
>5 y	122	1,710	7	0.99	
Born in Netherlands	117	2,437	5	0.65	
No information	37	615	6	0.83	
**Drug resistance**					
Susceptible	332	5,910	6	1	
H only	11	227	5	0.86	
S only	34	358	9	1.76	
Other patterns	26	221	12	2.24	
Multidrug-resistant	9	53	17	3.44	
Unknown	3	60	5	0.88	
**Multidrug resistance**					
Yes	9	53	17	3.21	2.64 (1.22 to 5.74)
No	406	6,776	6	1	1
**HIV infection**					
Yes	14	294	5	0.76	
No	401	6,535	6	1	

^a^RFLP, restriction fragment length polymorphism.

^b^Adjusted for year of diagnosis, age, nationality, and multidrug resistance

The association between the proportion of TB cases with the Beijing genotype and age was observed in particular in Dutch patients (chi square_trend_ 15.5; p<0.0001), and was not clear among non-Dutch patients (chi square_trend_ 0.8; p>0.2). Among Dutch patients, the Beijing genotype was more commonly found in new cases (6%, 133/2,130) than among retreated cases (4%, 18/498; p<0.05). Among non-Dutch patients, the Beijing genotype was slightly more common in retreated cases (8%, 19/229) than in new cases (6%, 204/3,218); however, this occurrence was not significant (p>0.2). The incidence pattern by person and time appears consistent with transmission of the Beijing genotype from immigrants to the Dutch population (*19*).

The proportion of TB cases with the Beijing genotype was not substantially associated with HIV infection, pulmonary localization, RFLP clustering, or duration of stay in the Netherlands (Table). The lack of association with RFLP-clustering suggests that the Beijing genotype is not spreading more quickly than other *M. tuberculosis* strains.

Of the nine patients with the Beijing genotype and multidrug resistance, four were from central and eastern Europe, three from Asia, one from Africa, and one from the Netherlands. One of these nine patients was RFLP-clustered; the other patient in that RFLP cluster had been diagnosed previously and had isolated isoniazid resistance.

During 1993 to 2000 in the Netherlands, the Beijing genotype represented 6% of TB cases. The Beijing genotype was associated with recent diagnoses, young age (in particular among Dutch citizens), nationality (Eastern Europe and Asia), and multidrug resistance. Although the Beijing genotype was associated with multidrug resistance, the number of MDRTB cases was small. No secondary cases were observed with the Beijing genotype and MDRTB. Of the nine cases of MDRTB, only one case may have been caused by transmission within the Netherlands from a case-patient who had isoniazid resistance at the start of treatment and may have acquired rifampicin resistance during treatment. However, the spread of the Beijing genotype among young people suggests the emergence of multidrug resistance and emphasizes the need for continued surveillance.
